# Analysis of the genomic sequence of *Philosamia cynthia* nucleopolyhedrin virus and comparison with *Antheraea pernyi* nucleopolyhedrin virus

**DOI:** 10.1186/1471-2164-14-115

**Published:** 2013-02-20

**Authors:** Heying Qian, Yuehua Zhang, Yejun Wu, Pingiang Sun, Shanying Zhu, Xijie Guo, Kun Gao, Anying Xu, Wenbing Wang

**Affiliations:** 1 Jiangsu University of Science and Technology, Sibaidu Road 1, Zhenjiang, 212018, China; 2 Jiangsu University, Xuefu Road 301, Zhenjiang, 212013, China; 3 The Sericultural Research Institute, Chinese Academy of Agricultural Sciences, Jiangsu University of Science and Technology, Sibaidu Road 1, Zhenjiang, 212018, China

## Abstract

**Background:**

Two species of wild silkworms, the Chinese oak silkworm (*Antheraea pernyi*) and the castor silkworm *Philosamia cynthia ricini*, can acquire a serious disease caused by Nucleopolyhedrin Viruses (NPVs) (known as AnpeNPV and PhcyNPV, respectively). The two viruses have similar polyhedral morphologies and their viral fragments share high sequence similarity. However, the physical maps of the viral genomes and cross-infectivity of the viruses are different. The genome sequences of two AnpeNPV isolates have been published.

**Results:**

We sequenced and analyzed the full-length genome of PhcyNPV to compare the gene contents of the two viruses. The genome of PhcyNPV is 125, 376 bp, with a G + C content of 53.65%, and encodes 138 open reading frames (ORFs) of at least 50 amino acids (aa) (GenBank accession number: JX404026). Between PhcyNPV and AnpeMNPV-L and -Z isolates, 126 ORFs are identical, including 30 baculovirus core genes. Nine ORFs were only found in PhcyNPV. Four genes, *cath*, *v-chi*, *lef 10* and *lef 11*, were not found in PhcyNPV. However, most of the six genes required for infectivity *via* the oral route were found in PhcyNPV and in the two AnpeNPV isolates, with high sequence similarities. The *pif-3* gene of PhcyNPV contained 59 aa extra amino acids at the N-terminus compared with AnpeNPV.

**Conclusions:**

Most of the genes in PhcyNPV are similar to the two AnpeNPV isolates, including the direction of expression of the ORFs. Only a few genes were missing from PhcyNPV. These data suggest that PhcyNPV and AnpeNPV might be variants of each other, and that the differences in cross-infection might be caused by gene mutations.

## Background

Baculoviridae is a large family of viruses that infect and kill insect species of different orders. Worldwide, they have been reported to infect over 600 host species [1], mostly from the order *Lepidoptera*. However, the viruses also infect insects from the orders *Diptera*, *Hymenoptera* and the crustacean order, *Decapoda*[2].

The complete genomes of 57 baculoviruses have been deposited in GenBank, including 41 *Alphabaculoviruses*, 12 *Betabaculoviruses*, three *Gammabaculoviruses* and one *Deltabaculovirus* genome [3]. Baculoviruses have been used extensively in many biological applications, for example as protein expression systems, as models of genetic regulatory networks and genome evolution, as putative nonhuman viral vectors for gene delivery, and as biological control agents against insect pests [4-6]. However, the diseases caused by baculoviruses are a major threat to the silk industry [3].

Silkmoths mostly belong to two families, the *Bombycidae* and *Saturniidae*, which secrete several varieties of silk fibers. The most common breeds are the domesticated silkworm (*Bombyx mori* L.) and the wild silkworms, including Chinese oak silkworm (*Antheraea pernyi* Guérin-Meneville), the castor silkworm (*Philosamia cynthia ricini*), the Indian tropical tasar silkworm (*A. mylitta* Drury) and the Japanese oak silkworm (*A. ayamamai* Guérin-Meneville) [7]. Silk production by these moths, especially *B*. *mori*, *A*. *pernyi* and *P*. *cynthia ricini*, are economically important worldwide. The domesticated silkworm (*B*. *mori*) has been used for silk production by Chinese farmers for approximately 5000 years [7]. It has since spread to Korea, Japan, India, Brazil and the rest of the world. The most well-known species among wild silkworms is the Chinese oak silkworm (*A*. *pernyi*). It is commercially cultivated for silk production, primarily in China, India and Korea [8]. This silkworm species and the castor silkworm (*P. Cynthia ricini*) were introduced into China for silk production in the 1950s [7].

These three species can be infected by baculoviruses: *B. mori* Nucleopolyhedrosis virus (BmNPV), *A*. *pernyi* NPV (AnpeNPV) and *P*. *Cynthia ricini* NPV (PhcyNPV) according to their respective hosts [9-12]. The complete sequence of BmNPV strain T3 (GenBank: NC_001962) was published in 1999, and the sequence of *B. mandarina* NPV (BomaNPV, a variant of BmNPV) was published in 2010 (GenBank: FJ882854) [13,14]. Two AnpeNPV isolates were published in 2007 (GenBank: EF207986 and NC_008035). We compared AnpeNPV and PhcyNPV, and found that these two viruses have similar polyhedral morphologies and their viral fragments show high sequence similarity. However, the physical maps of the viral genomes and cross-infectivity of the viruses are different. Therefore, we analyzed the whole genome sequence of PhcyNPV to obtain more information about the viral genes related to infection.

## Results

### Sequencing, assembly, and analysis of the PhcyNPV genome

The entire PhcyNPV dsDNA genome was sequenced and assembled into a contiguous sequence of 125, 376 bp, with a G + C content of 53.65% (GenBank: JX404026). Many baculoviruses have an approximate GC content of 41%, whereas PhcyNPV and several other baculoviruses have significantly higher values (50.1% for CfMNPV, 50.9% for CuniNPV, 53.5% for AnpeNPV-L2, 53.5% for AnpeNPV-Z, 53.5% for LyxyNPV, 55.1% for OpMNPV and 57.5% for LdMNPV). However, a detailed analysis of DNA content did not show a clear pattern of GC content that could be associated with each genus [7].

**Figure 1:**
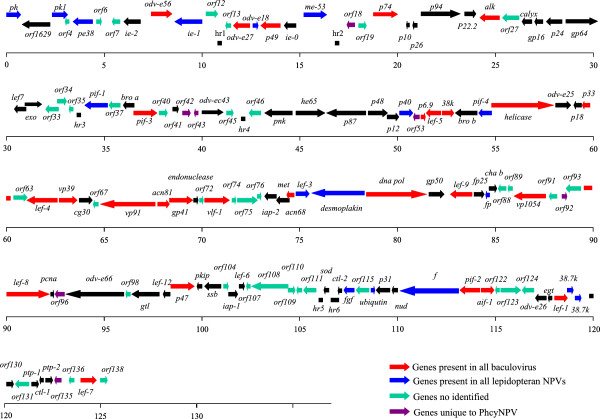
Linear map of the 138 predicted ORFs for the complete PhcyNPV genome.

One hundred and thirty-eight ORFs were identified in the PhcyNPV genome that encoded putative proteins of at least 50 amino acids (aa) with minimal adjacent ORF overlap. Designating the polyhedrin gene as the first ORF and noting its clockwise orientation, 65 ORFs were identified in the clockwise orientation, and 76 were identified in the counter-clockwise orientation. A BLAST analysis of PhcyNPV ORFs showed that 94.9% (131/138) had assigned functions or homologs in other baculovirus genomes (Figure 1 and Additional file 1: Table S1). Seven unique ORFs were identified (nos. 18, 42, 43, 53, 92, 96, and 135), which putatively encoded polypeptides from 68 to 223 aa. However, the directions of transcription of these genes are different, and it is not known if they are expressed.

### Comparison of the PhcyNPV genome with the genomes of two AnpeNPV isolates

**Figure 2:**
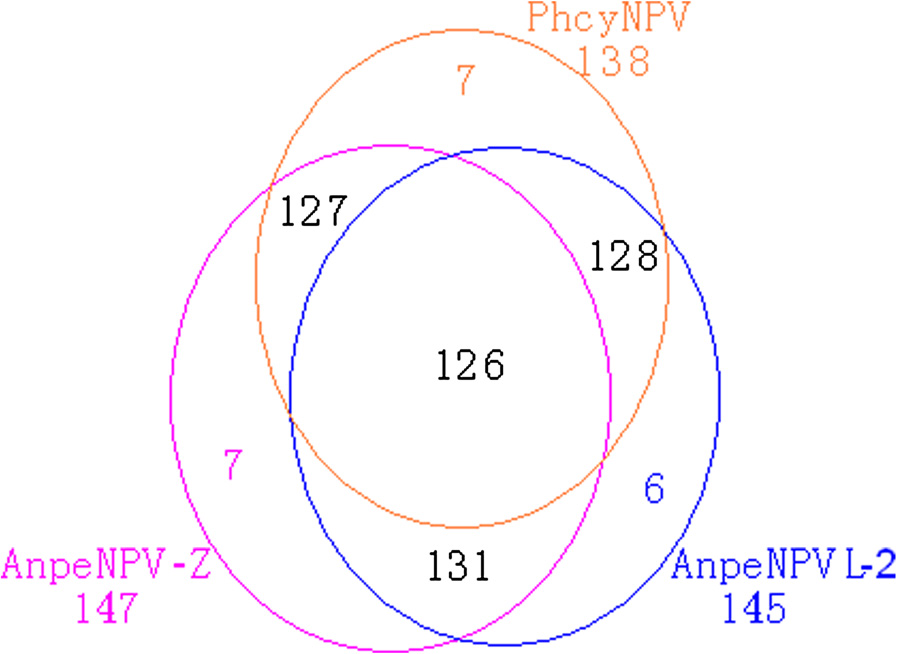
**Comparison of the genomes of three types of baculoviruses, PhcyNPV, AnpeNPV-L2 and AnpeNPV-Z.** The different circles represent the genes of each type of virus. The numbers contained within the overlapping regions indicate the numbers of shared genes, and the numbers within the circles, but outside the overlapping regions, indicate the number of unique genes in each genome.

Most genes in the PhcyNPV genome are similar to the previously published sequences of AnpeNPV, -L2 isolate (126, 246 bp, GenBank: EF207986) and -Z isolate (126, 630 bp, GenBank: NC_008035). One hundred and twenty-six ORFs are identical between PhcyNPV and the AnpeMNPV-L2 and -Z isolates, including 30 baculovirus core genes (Figure 1). Some ORFs appear to be present only in one of the isolates: nine ORFs in PhcyNPV, six in AnpeNPV-L2 and seven in AnpeNPV-Z (Figure 2). Comparing the three viral genomes, the main changes of the ORFs were found in five regions. In the region of 30, 622 to 32, 874 nt of PhcyNPV, two genes, *cath* and *v-chi*, are missing in PhcyNPV. Indels in the coding regions of five ORFS caused their lengths to vary. In region 38, 639 to 39, 754 nt, there are three ORFs (Phcy ORF41 - 43) in PhcyNPV that are not present in AnpeNPV-L2. Conversely, there are three ORFs in AnpeNPV-L2 with lengths of 82 (Anpe-ORF044), 56 (Anpe-ORF045), and 92 aa (*chtB* 2) in this region. Interestingly, in this region, AnpeNPV-Z has a homolog of PhcyNPV ORF41 (132 aa) with a length of 71 aa. Homologous genes for PhcyNPV ORF41 were also found in some other baculoviruses, such as *Choristoneura fumiferana* DEF MNPV (Cf DEF MNPV, orf107, 135 aa), *Hyphantria cunea* NPV (HycuNPV, ORF43, 135aa) and *Anticarsia gemmatalis* NPV (AngeNPV, AGNV_gp111, 135aa). Although Anpe-ORF044 and Anpe-ORF045 were found in AnpeNPV-Z, the *chtB*-2 gene was missing in this region. Additionally, the *p12* and Anpe-ORF069 genes were found in the PhcyNPV genome and the AnpeNPV L2 isolate, but both genes were missing in AnpeNPV-Z. In the PhcyNPV genome, *lef 10* and *lef 11* could not be identified.

Certain ORFS were truncated or extended. For example, the *p94* (*Ac 134*) genes in AnpeNPV-L2 and in PhcyNPV were predicted to encode proteins of 492 aa and 795 aa, respectively, whereas, in the –Z isolate, the gene could be divided into two ORFs (390 aa and 99 aa). Homologs of *p94* are present in the genomes of most Group I baculoviruses. They are also present in some members of Group II and GV, and in several polydnaviruses. Disruption of the *p94* gene had no effect on the ability of AcMNPV to infect *S. frugiperda* larvae by either the oral or intrahemocelic route [15]. Interestingly, the *egt* genes of these three viruses are different; the ORF of PhcyNPV *egt* comprises 87 aa in the EGT N-terminus of AcMNPV, similar to AnpeNPV (−L2, 132 aa; -Z, 79 aa). The *egt* gene encodes ecdysteroid UDP-glucosyltransferase (EGT), and homologs are found in all Groups I, II and most GV genomes, mostly encoding proteins of 400 to 500 aa. The function of EGT is to block molting and pupation in infected larvae by catalyzing the transfer of glucose from UDP-glucose to ecdysteroids, thereby inactivating these insect molting hormones [16,17]. Deletion of the *egt* gene from the genome of AcMNPV caused the virus to kill the insects more quickly [18]. The day after *P*. *cynthia ricini* larvae were infected with PhcyNPV or AnpeNPV by *per os* inoculation, nearly one-third of the insects died. This might have been caused by *egt* mutation. The observation requires further investigation.

### Predicting cellular location of the PhcyNPV genes

Viral proteins that contain a signal peptide are usually involved in viral particle packaging or interaction with hosts. To determine which genes might be related to host interaction, we predicted the presence of a signal peptide in the proteins encoded by all the ORFs of PhcyNPV. Twenty-three gene products of PhcyNPV were identified that might be located in the endoplasmic reticulum (ER). Most of the proteins were found to function in viral packaging and interaction with hosts (Table 1). Among the 23 genes, six are unique to the three NPVs compared in this paper, and one unknown gene is unique to PhcyNPV. The fusion (*f*) gene was the only gene whose protein product showed a low probability of being located in the ER (prediction value 0.33). We analyzed the *f* homolog genes from other baculoviruses and they appear to be divided into two groups: those with high identities (prediction value 0.7, such as AngeNPV, GenBank: YP_803416) and those with low possibilities (prediction value 0.3, AcMNPV, GenBank: NP_054052) (data not shown).

**Table 1:** PhcyNPV ORFs with a predicted subcellular location in the ER (endoplasmic reticulum)

**Gene number**	**ORF (aa)**	**Gene name**	**Prediction value**
PhcyNPV012	95	chitin-binding protein 1	0.99
PhcyNPV028	102	gp16	0.99
PhcyNPV030	509	gp64	0.94
PhcyNPV036	531	pif-1	0.99
PhcyNPV053	99	**Phcy-ORF053**	0.58
PhcyNPV058	172	pif-4/19 k/odv-e28	0.99
PhcyNPV060	228	odv-e25	0.99
PhcyNPV061	158	p18	0.90
PhcyNPV063	191	Anpe-ORF064	0.99
PhcyNPV074	84	Anpe-ORF077	0.99
PhcyNPV083	362	gp50 spindlin	0.99
PhcyNPV088	167	Anpe-ORF093	0.90
PhcyNPV091	146	Anpe-ORF098	0.61
PhcyNPV097	680	odv-e66	0.99
PhcyNPV113	63	ctl 2	0.83
PhcyNPV114	185	fgf	0.92
PhcyNPV115	270	Anpe-ORF123	0.99
PhcyNPV119	654	F protein	0.33 *
PhcyNPV120	382	pif-2	0.99
PhcyNPV121	324	arif-1	0.99
PhcyNPV126	87	egt	0.99
PhcyNPV131	336	Anpe-ORF139	0.91
PhcyNPV133	53	ctl 1	0.99

*Low probability of an ER location; bold face indicates an ORF found only in the PhcyNPV genome.

We also identified 19 gene products that might be located in the mitochondria (Table 2). To date, few baculovirus genes have been found to interact with the mitochondria, an important organ involved in energy metabolism and apoptosis. Among the 19 genes, 11 genes have high prediction values (more than 0.5), and eight genes have low prediction values (less than 0.5). Interestingly, eight genes are unique to the three compared NPVs; five of them are unique to PhcyNPV.

**Table 2:** PhcyNPV ORFs with a predicted mitochondrial location

**Gene number**	**ORF (aa)**	**Gene name**	**Prediction value**
PhcyNPV006	69	Anpe-ORF006	0.60
PhcyNPV018	82	**Phcy-ORF018**	0.41 *
PhcyNPV025	465	alkaline exonuclease	0.24 *
PhcyNPV032	285	EXO III v-trex	0.92
PhcyNPV039	264	pif-3	0.67
PhcyNPV042	109	**Phcy-ORF042**	0.21 *
PhcyNPV043	68	**Phcy-ORF043**	0.83
PhcyNPV050	387	P48	0.22 *
PhcyNPV054	79	p 6.9	0.83
PhcyNPV056	313	38 K	0.73
PhcyNPV062	253	p33	0.38 *
PhcyNPV069	214	AcN81	0.25 *
PhcyNPV071	98	endonuclease	0.45 *
PhcyNPV078	264	methyl transferase	0.56
PhcyNPV090	376	vp1054	0.37 *
PhcyNPV092	117	**Phcy-ORF092**	0.68
PhcyNPV096	223	**Phcy-ORF096**	0.54
PhcyNPV098	157	Anpe-ORF105	0.68
PhcyNPV136	149	Anpe-ORF143	0.70

*Low probability of a mitochondrial location; bold face indicates an ORF found only in the PhcyNPV genome.

Of the eight unique predicted genes of PhcyNPV, five might be related to energy metabolism and/or apoptosis, and one is located in ER. These data imply that the unique genes of baculovirus mostly determine their interaction with individual insect breeds.

### The genes related to oral infectivity

A previous report showed that the cross-infectivity characteristics of PhcyNPV and AnpeNPV were different. AnpeNPV caused 57% mortality in larvae of *P. cynthia rici*, whereas PcrNPV did not kill the larvae of *A. pernyi*[19]. Hence, the six genes (*pif* 0 to 5) required for infectivity via the oral route were analyzed. Most of them are very similar between PhcyNPV and the two AnpeNPV isolates, except for PIF-3, which in PhcyNPV has a 59aa extra amino acid sequence in the N-terminus.

## Discussion

**Figure 3:**
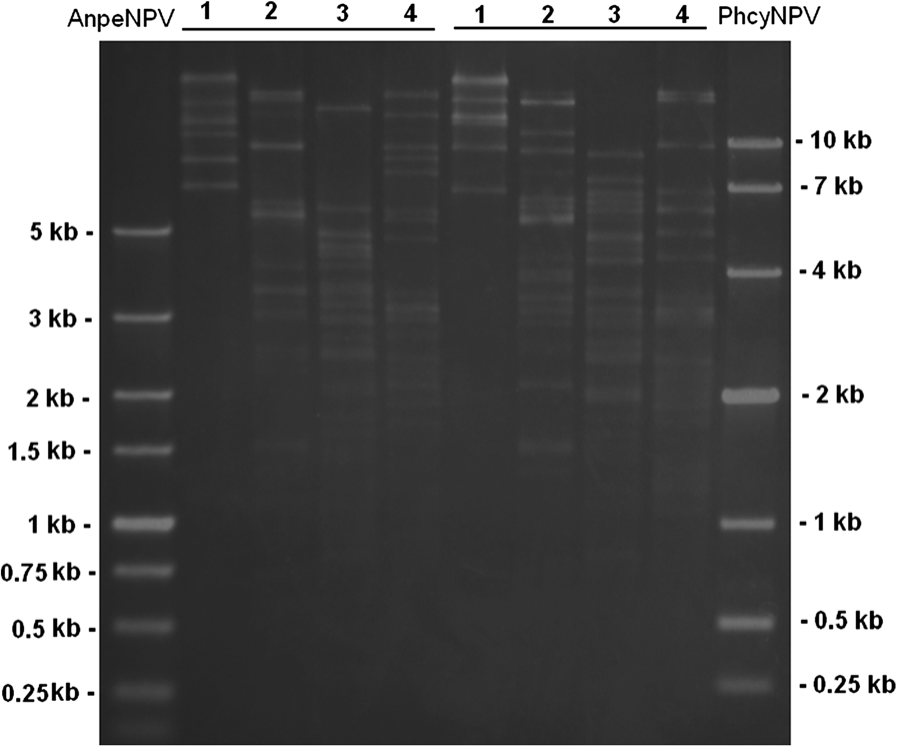
**Viral DNAs of *Philosamia cynthia *nucleopolyhedrovirus (PhcyNPV) and *Antheraea pernyi *NPV (AnpeNPV) digested with restriction endonucleases.** 1, *Eco*R I; 2, *Hin*d III; 3, *Pst* I; 4, *Sal* I.

Improvements in sequencing technology have made the analysis of the genome sequence of a baculovirus easier. However, purifying a type of baculovirus is not easy if a permissive insect cell line for the viral infection has not been established. At present, no permissive line for PhcyNPV is available. We collected budded virus (BV) of PhcyNPV from larvae infected by oral inoculation, and attempted to infect some common insect cell lines, such as BmN, Sf9, Tn5, and Spli cells; however, no symptoms of infection were observed. We then inoculated the *P. cynthia rici* pupae using the BV obtained, and seeded the infected hemocyte cells on the plate containing insect medium with soft agar gel (0.5%). After 3–5 days of culture, when polyhedra were observed in the cells, the gel was aspirated and injected into healthy pupae. After several repeats, we obtained purified PhcyNPV. The DNA was digested with restriction endonucleases and showed clear bands (Figure 3).

We observed that PhcyNPV was not infectious to *A. pernyi* by oral inoculation. This might involve the oral infection factors, including *p74/ pif-0*[20], *pif-1*[21], *pif-2*[22], *pif-3*[23], *pif-4/19 k/odv-e28*[24], and *pif-5/odv-e56*[25,26]. These six genes are all structural components of occlusion derived viruses (ODVs). P74 mediates the specific binding of ODVs to primary cellular targets in the midgut epithelia [20], while pif-3 appears to mediate another crucial, but as yet unidentified, event during primary infection [23]. The proteins encoded by *pif-4* and *pif-5* have essential roles in the *per os* infection route [24-26]. Most of these genes are highly similar to those of AnpeNPV, except for PIF-3 of PhcyNPV, which has an extra 59-aa N-terminal structure. However, no homologous structure was found in other viruses or species. The PIF-3 proteins from other baculoviruses are all about 200 aa, and show low levels of sequence similarity to each other. Interestingly, the C-terminal structure of PhcyNPV PIF-3 is similar to that of a membrane-spanning Ca-ATPase from the fungus *Spathaspora passalidarum* (GenBank: EGW31824), and an ABC transporter exported protein from the bacterium *Pseudomonas* sp. R81 (GenBank: ZP_11190693). These proteins are associated with the cell membrane. These observations imply that baculovirus PIF-3 might be related to the viral trans-membrane process.

The hrf1 gene from LdMNPV expands the host range of AcMNPV both *in vitro* and *in vivo*, allowing it to infect non-permissive hosts [27,28]. The only baculovirus homolog of this gene is found in OpMNPV. Two conotoxin-like (*ctl*) genes, *ctl1* and *ctl2*, are also found in both of these genomes. Other baculoviruses encode one or the other, but only OpMNPV and LdMNPV encode both. A report indicated that there is a clear phylogenetic link between *hrf1*and the presence of both *ctl* genes [29]. We also found the two *ctl* genes in the genomes of PhcyNPV and AnpeNPV. However, the *hrf1* gene was missing. It seems that functions of *ctl1* and *ctl2* are distinct when appearing with *hrf1*.

## Conclusions

Most of the genes in PhcyNPV were similar to the two AnpeNPV isolates, including the direction of expression of the ORFs. Only a few genes were missing in PhcyNPV. These data suggest that PhcyNPV and AnpeNPV might be variants with each other, and the difference of cross-infection might be caused by gene mutations.

## Methods

### Viruses

The Institute of Guangxi Sericultural Research and Development in South China provided the occlusion bodies (OBs) of PhcyNPV. The AnpeNPV was kindly provided by Professor Qin Li of Shenyang Agricultural University in north China.

### Viral DNA preparation and sequencing

The procedures for isolating OBs and preparing viral DNA were as described by Cheng et al., 2005 [30]. In brief, the OBs were purified by density gradient centrifugation, and were dissociated with a lysis buffer containing 0.1 mol/L Na_2_CO_3_ and 0.15 mol/L NaCl on ice for 30 min. After that, 0.5% SDS and proteinase K (50 mg/mL) were added and incubated at 37°C for 4 h. The digested solution was progressively extracted with phenol and chloroform mixtures. DNA was precipitated with 70% ethanol. The DNA was dried and dissolved in 2.0 mmol/L Tris (pH 8.0). The quantity and quality of the isolated DNA were determined spectrophotometrically and by electrophoresis on 0.7% agarose gel. A DNA fragment library of PhcyNPV was constructed using the shotgun method described by Zhu et al. [31]. All clones were sequenced and the full-length sequence was constructed by the Chinese National Human Genome Center at Beijing.

### Sequence analysis

ORFs in the PhcyNPV genome were identified using ORF finder (http://www.ncbi.nlm.nih.gov/gorf/gorf.html). All BLAST searches were done through the National Center for Biotechnology Information (NCBI) websites. The signal peptide data were downloaded from http://www.cbs.dtu.dk/services/SignalP/ using the software Prodotar v.1.03.

## Competing interests

There are no competing interests, including financial competing interests, in this paper.

## Author’ contributions

AX and WW conceived the project and drafted the final manuscript. HQ and YZ carried out the molecular genetic studies. PS and XG participated in its design and coordination and helped to draft the manuscript. HQ and KG participated in the design of the study. HQ, YW and SZ participated in the sequence alignment. All authors read and approve the final manuscript.

## Supplementary Material

Additional file 1: Table S1 Predicted ORFs in the PhcyNPV genome, comparing to the closely related AnpeNPV. Click here for file
