# Supplementary material for: Analysis of the genomic sequence of Philosamia cynthia nucleopolyhedrin virus and comparison with Antheraea pernyi nucleopolyhedrin virus
Source: BMC Genomics. 2013 Feb 20;14:115. doi: 10.1186/1471-2164-14-115 (PMC3600015; doi:10.1186/1471-2164-14-115)
Supplement: Additional file 1: Table S1 — Predicted ORFs in the PhcyNPV genome, comparing to the closely related AnpeNPV. [file 1471-2164-14-115-S1.doc]

Table 1 Predicted ORFs in the PhcyNPV genome, comparing to the closely related AnpeNPV.

| **ORF** |  | **ORF Lenth** | **Position** | | | **MininGene Annotation Pipeline** | **AnpeNPV -L2** | **AnpeNPV Z** |
| --- | --- | --- | --- | --- | --- | --- | --- | --- |
|  | **ORF aa** |  | **Start** | **End** | **Strand** |  | **ORF aa** | **ORF aa** |
| PhcyNPV001 | 245 | 738 | 1 | 738 | + | polyhedrin | 245 |  |
| PhcyNPV002 | 591 | 1776 | 735 | 2510 | - | 1629 | 591 |  |
| PhcyNPV003 | 267 | 804 | 2509 | 3312 | + | protein kinase 1 | 267 |  |
| PhcyNPV004 | 76 | 231 | 3265 | 3495 | - | Anpe-ORF004 | 76 |  |
| PhcyNPV005 | 301 | 906 | 3531 | 4436 | - | PE 38 | 302 |  |
| PhcyNPV006 | 69 | 210 | 4525 | 4734 | + | Anpe-ORF006 | 56 |  |
| PhcyNPV007 | 145 | 438 | 5220 | 5657 | - | Anpe-ORF007 | 145 |  |
| PhcyNPV008 | 356 | 1071 | 5774 | 6844 | - | ie-2 | 295, 84 |  |
| **PhcyNPV009** | 374 | 1125 | 7416 | 8540 | + | odv-e56/pif-5 | 374 |  |
| PhcyNPV010 | 485 | 1458 | 8892 | 10349 | - | ie-1 | 590 | 577 |
| PhcyNPV011 | 189 | 570 | 10402 | 10971 | + | Anpe-ORF012 | 189 |  |
|  |  |  | 11029 | 11159 |  | hr1 |  |  |
| PhcyNPV012 | 95 | 288 | 11274 | 11561 | - | chitin-binding protein 1 | 95 |  |
| **PhcyNPV013** | 296 | 891 | 11564 | 12454 | - | odv-e27 | 296 | 308 |
| **PhcyNPV014** | 87 | 264 | 12470 | 12733 | + | odv-e18 | odv-e18 | 68 |
| **PhcyNPV015** | 468 | 1407 | 12773 | 14179 | - | P49 | 482 |  |
| PhcyNPV016 | 240 | 723 | 14189 | 14911 | - | ie-0 | 240 |  |
| PhcyNPV017 | 477 | 1434 | 15161 | 16594 | + | ME53 | 467 | 372 |
|  |  |  | 16723 | 17334 |  | hr2 |  |  |
| PhcyNPV018 | 82 | 249 | 17214 | 17462 | - | Phcy-ORF018 |  |  |
| PhcyNPV019 | 165 | 498 | 17461 | 17958 | + | Anpe-ORF019 | 247 |  |
| **PhcyNPV020** | 640 | 1923 | 18297 | 20219 | + | P74/pif-0 | 640 |  |
| PhcyNPV021 | 86 | 261 | 20216 | 20476 | - | P10 | 87 |  |
| PhcyNPV022 | 236 | 711 | 20519 | 21229 | - | P26 | 240 |  |
| PhcyNPV023 | 795 | 2388 | 21297 | 23684 | + | P94-like protein | 492 | 390+ 99 |
| PhcyNPV024 | 209 | 630 | 23800 | 24429 | + | p22.2 | 209 |  |
| **PhcyNPV025** | 465 | 1398 | 24456 | 25853 | - | alkaline exonuclease | 465 |  |
| PhcyNPV026 | 223 | 672 | 25736 | 26407 | - | Anpe-ORF027 | 224 | 75 |
| PhcyNPV027 | 289 | 870 | 26408 | 27277 | - | polyhedral calyx protein | 289 |  |
| PhcyNPV028 | 102 | 309 | 27325 | 27633 | - | gp16 | 102 |  |
| PhcyNPV029 | 190 | 573 | 27646 | 28218 | - | viral capsid protein p24 | 188 | 190 |
| PhcyNPV030 | 509 | 1530 | 28657 | 30186 | + | gp64 | 509 , cathepsin 324 , v-chi 552 , **(A)** |  |
| PhcyNPV031 | 154 | 465 | 30622 | 31026 | - | lef-7 | 210 |  |
| PhcyNPV032 | 285 | 858 | 30860 | 31717 | + | EXO III v-trex | 230 |  |
| PhcyNPV033 | 242 | 729 | 31803 | 32531 | - | Anpe-ORF035 | 236 |  |
| PhcyNPV034 | 231 | 696 | 32179 | 32874 | + | Anpe-ORF036 | 225 |  |
| PhcyNPV035 | 83 | 252 | 33024 | 33275 | - | Anpe-ORF037 | 83 |  |
|  |  |  | 33310 | 33441 |  | hr3 |  |  |
| **PhcyNPV036** | 531 | 1596 | 33439 | 35034 | - | pif-1 | 531 |  |
| PhcyNPV037 | 234 | 705 | 35076 | 35780 | - | Anpe-ORF039 | 236, 81 |  |
| PhcyNPV038 | 176 | 531 | 36081 | 36611 | - | bro a | 177 |  |
| **PhcyNPV039** | 264 | 795 | 36460 | 37254 | + | pif-3 | 205 |  |
| PhcyNPV040 | 416 | 1251 | 37263 | 38513 | + | Anpe-ORF043 | 416, 82, 56, chtB2 92, **(B)** | 416, 71, 82, 56 |
| PhcyNPV041 | 132 | 399 | 38639 | 39037 | + | similar to Cf DEFNPV ORF107 | missing | 71 |
| PhcyNPV042 | 109 | 330 | 39094 | 39423 | + | Phcy-ORF042 |  |  |
| PhcyNPV043 | 68 | 207 | 39548 | 39754 | - | Phcy-ORF043 |  |  |
| PhcyNPV044 | 391 | 1176 | 39942 | 41117 | + | odv-ec43 | 391 |  |
| PhcyNPV045 | 102 | 309 | 41120 | 41428 | + | Anpe-ORF048 | 102 |  |
|  |  |  | 41438 | 41707 |  | hr4 |  |  |
| PhcyNPV046 | 252 | 759 | 41699 | 42457 | - | Anpe-ORF049 | 251 |  |
| PhcyNPV047 | 680 | 2043 | 42547 | 44589 | - | pnk/pnl | 680 |  |
| PhcyNPV048 | 575 | 1728 | 44784 | 46511 | + | HE65 | 553 |  |
| PhcyNPV049 | 637 | 1914 | 46593 | 48506 | - | P87 | 637 | 650 |
| PhcyNPV050 | 387 | 1164 | 48530 | 49693 | + | P48 | 387 |  |
| PhcyNPV051 | 113 | 342 | 49686 | 50027 | + | P12 | 113 | missing |
| **PhcyNPV052** | 241 | 726 | 50024 | 50749 | + | P40/odv-nc42 | 355 | 354 |
| PhcyNPV053 | 99 | 300 | 50721 | 51020 | - | Phcy-ORF053 |  |  |
| **PhcyNPV054** | 79 | 240 | 51130 | 51369 | + | p 6.9 | 79 |  |
| **PhcyNPV055** | 259 | 780 | 51366 | 52145 | - | lef-5 | 259 |  |
| **PhcyNPV056** | 313 | 942 | 52092 | 53033 | + | 38K | 313 |  |
| PhcyNPV057 | 339 | 1020 | 53048 | 54067 | - | bro b | 339 |  |
| **PhcyNPV058** | 172 | 519 | 54117 | 54635 | - | pif-4/19k/odv-e28 | 172 |  |
| **PhcyNPV059** | 1212 | 3639 | 54625 | 58263 | + | helicase | 1212 |  |
| PhcyNPV060 | 228 | 687 | 58303 | 58989 | - | odv-e25 | 228 |  |
| PhcyNPV061 | 158 | 477 | 58994 | 59470 | - | p18 | 158 |  |
| **PhcyNPV062** | 253 | 762 | 59469 | 60230 | + | p33 | 253 |  |
| PhcyNPV063 | 191 | 576 | 60244 | 60819 | + | Anpe-ORF064 | 204 | 211 |
| **PhcyNPV064** | 458 | 1377 | 60806 | 62182 | - | lef-4 | 458 |  |
| **PhcyNPV065** | 335 | 1008 | 62195 | 63202 | + | vp39 | 337 |  |
| PhcyNPV066 | 242 | 729 | 63208 | 63936 | + | cg30 | 242 |  |
| PhcyNPV067 | 67 | 204 | 64350 | 64553 | - | Anpe-ORF069 | 67 | missing |
| **PhcyNPV068** | 863 | 2592 | 64907 | 67498 | - | vp91 | 855 |  |
| **PhcyNPV069** | 214 | 645 | 67714 | 68358 | + | AcN81 | 214 | 152, 214 |
| **PhcyNPV070** | 362 | 1089 | 68351 | 69439 | + | gp41 | 362 |  |
| PhcyNPV071 | 98 | 297 | 69439 | 69735 | + | endonuclease | 98 |  |
| PhcyNPV072 | 103 | 312 | 69738 | 70049 | + | Anpe-ORF075 | 103 |  |
| **PhcyNPV073** | 370 | 1113 | 70054 | 71166 | + | vlf-1 | 370 |  |
| PhcyNPV074 | 84 | 255 | 71178 | 71432 | + | Anpe-ORF077 | 84 |  |
| PhcyNPV075 | 311 | 936 | 71434 | 72369 | + | Anpe-ORF078 +Anpe-ORF079 | 130, 176 | 173， 130 |
| PhcyNPV076 | 85 | 258 | 72366 | 72623 | + | Anpe-ORF080 | 85, 57 |  |
| PhcyNPV077 | 242 | 729 | 72836 | 73564 | - | iap 2 | 242 | 189 |
| PhcyNPV078 | 264 | 795 | 73545 | 74339 | - | putative methly transferase | 264 | 263 |
| PhcyNPV079 | 136 | 411 | 74290 | 74700 | - | AcN68 | 136 |  |
| PhcyNPV080 | 351 | 1056 | 74702 | 75757 | + | lef 3 | 369 |  |
| **PhcyNPV081** | 821 | 2466 | 75810 | 78275 | - | desmoplakin | 825 | 829 |
| **PhcyNPV082** | 956 | 2871 | 78285 | 81155 | + | DNA polymerase | 956 | 920 |
| PhcyNPV083 | 362 | 1089 | 81215 | 82303 | + | gp50 spindlin | 364 |  |
| **PhcyNPV084** | 495 | 1488 | 82331 | 83818 | - | lef-9 | 495 | 492 |
| PhcyNPV085 | 209 | 630 | 83867 | 84496 | + | Fusion protein-like/25FP | 209 |  |
| PhcyNPV086 | 84 | 255 | 84653 | 84907 | + | FP protein | 83 |  |
| PhcyNPV087 | 156 | 471 | 84879 | 85349 | + | ChaB | 156 | 154 |
| PhcyNPV088 | 167 | 504 | 85375 | 85878 | - | Anpe-ORF093 | 177, 77 | 177, 143 |
| PhcyNPV089 | 114 | 345 | 86549 | 86893 | - | Anpe-ORF095 | 69 |  |
| **PhcyNPV090** | 376 | 1131 | 86955 | 88085 | - | vp1054 | 376, lef10 81, **(C)** |  |
| PhcyNPV091 | 146 | 441 | 88148 | 88588 | - | Anpe-ORF098 | 146 |  |
| PhcyNPV092 | 117 | 354 | 89003 | 89356 | + | Phcy-ORF092 |  |  |
| PhcyNPV093 | 199 | 600 | 89215 | 89814 | - | Anpe-ORF099 | 319 |  |
| **PhcyNPV094** | 873 | 2622 | 89838 | 92459 | + | lef-8 | 873 | 874 |
| PhcyNPV095 | 72 | 219 | 92495 | 92713 | + | PCNA | 246 |  |
| PhcyNPV096 | 223 | 672 | 92767 | 93438 | - | Phcy-ORF096 | 110, 89, **(D)** | 222 |
| PhcyNPV097 | 680 | 2043 | 93647 | 95689 | - | odv-e66 | 679 |  |
| PhcyNPV098 | 157 | 474 | 95764 | 96237 | - | Anpe-ORF105 | 129, 70 |  |
| PhcyNPV099 | 495 | 1488 | 96320 | 97807 | - | global transactivator-like protein | 495 |  |
| PhcyNPV100 | 171 | 516 | 97816 | 98331 | - | lef-12 | 171 |  |
| **PhcyNPV101** | 399 | 1200 | 98315 | 99514 | + | transcription regulator p47 | 399 |  |
| PhcyNPV102 | 166 | 501 | 99533 | 100033 | - | pkip | 166 |  |
| PhcyNPV103 | 300 | 903 | 100042 | 100944 | - | ssDNA binding protein | 300 | 300 |
| PhcyNPV104 | 129 | 390 | 100997 | 101386 | + | Anpe-ORF112 | 129 |  |
| PhcyNPV105 | 280 | 843 | 101383 | 102225 | + | iap 1 | 280 |  |
| PhcyNPV106 | 177 | 534 | 102075 | 102608 | + | lef-6 | 127 | 106 |
| PhcyNPV107 | 67 | 204 | 102638 | 102841 | - | Anpe-ORF115 | 67 |  |
| PhcyNPV108 | 447 | 1344 | 102897 | 104240 | - | Anpe-ORF116 | 447 |  |
| PhcyNPV109 | 185 | 558 | 104239 | 104796 | + | Anpe-ORF117 | 185 |  |
| PhcyNPV110 | 131 | 396 | 104865 | 105260 | - | Anpe-ORF118 | 164 | 128 |
| PhcyNPV111 | 234 | 705 | 105270 | 105974 | - | Anpe-ORF119 | 234 |  |
|  |  |  | 106083 | 106141 |  | hr 5 |  |  |
| PhcyNPV112 | 150 | 453 | 106139 | 106591 | - | sod | 150 |  |
|  |  |  | 106633 | 106883 |  | hr 6 |  |  |
| PhcyNPV113 | 63 | 192 | 106924 | 107115 | - | ctl 2 | 63 |  |
| PhcyNPV114 | 185 | 558 | 107210 | 107767 | - | fgf | 185 |  |
| PhcyNPV115 | 270 | 813 | 107943 | 108755 | - | Anpe-ORF123 | 270 |  |
| PhcyNPV116 | 76 | 231 | 108552 | 108782 | + | ubiqutin | 76 |  |
| PhcyNPV117 | 290 | 873 | 108866 | 109738 | - | p31 | 290, lef11 108, **(E)** |  |
| PhcyNPV118 | 205 | 618 | 109907 | 110524 | - | NUDIX hydrolase | 205 |  |
| PhcyNPV119 | 654 | 1965 | 110659 | 112623 | - | F protein | 654 |  |
| **PhcyNPV120** | 382 | 1149 | 112812 | 113960 | - | pif-2 | 382 |  |
| PhcyNPV121 | 324 | 975 | 113981 | 114955 | + | arif-1 | 324 |  |
| PhcyNPV122 | 103 | 312 | 115187 | 115498 | - | Anpe-ORF131 | 103 |  |
| PhcyNPV123 | 353 | 1062 | 115500 | 116561 | + | Anpe-ORF132 | 353 |  |
| PhcyNPV124 | 206 | 621 | 116598 | 117218 | - | Anpe-ORF133 | 206 |  |
| PhcyNPV125 | 197 | 594 | 117187 | 117780 | - | odv-e26 | 197 |  |
| PhcyNPV126 | 87 | 264 | 117949 | 118212 | - | egt | 132 | 79 |
| **PhcyNPV127** | 243 | 732 | 118323 | 119054 | + | lef-1 | 243 |  |
| PhcyNPV128 | 149 | 450 | 119012 | 119461 | + | 38.7k (N-terminal) | 307, **(F)** | 307 |
| PhcyNPV129 | 166 | 501 | 119434 | 119934 | + | 38.7k (C-terminal) |  |  |
| PhcyNPV130 | 172 | 519 | 119992 | 120510 | + | similar to CfMNPVgpORF11 | 124 | 121 |
| PhcyNPV131 | 336 | 1011 | 120593 | 121603 | - | Anpe-ORF139 | 336 |  |
| PhcyNPV132 | 178 | 537 | 121724 | 122260 | + | ptp 1 | 178 |  |
| PhcyNPV133 | 53 | 162 | 122238 | 122399 | - | ctl 1 | 53 |  |
| PhcyNPV134 | 160 | 483 | 122528 | 123010 | + | ptp 2 | 160 |  |
| PhcyNPV135 | 137 | 414 | 123073 | 123486 | - | PhcyNPV135 |  |  |
| PhcyNPV136 | 149 | 450 | 123522 | 123971 | - | Anpe-ORF143 | 149 |  |
| **PhcyNPV137** | 246 | 741 | 124110 | 124850 | + | lef-2 | 246 | 246, 145, **(G)** |
| PhcyNPV138 | 134 | 405 | 124890 | 125294 | + | Anpe-ORF145 | 128 | 128 |

The 30 baculovirus core genes are in bold.

1. In PhcyNPV, the *cath* and *v-chi* were missing
2. Here, there was a big difference between the three type viruses, in AnpeNPV-Z, a *cht B* gene was lost
3. Here, the *lef* 10 gene was missing in PhcyNPV
4. Here, there was a big difference between the three type viruses
5. The *lef* 11 gene was missing in PhcyNPV
6. In PhcyNPV, the gene was divided into two genes.
7. Here, There was a unique ORF with 145 aa in AnpeNPV-Z
